# Radical Scavenging Activity-Based and AP-1-Targeted Anti-Inflammatory Effects of Lutein in Macrophage-Like and Skin Keratinocytic Cells

**DOI:** 10.1155/2013/787042

**Published:** 2013-03-07

**Authors:** Jueun Oh, Ji Hye Kim, Jae Gwang Park, Young-Su Yi, Kye Won Park, Ho Sik Rho, Min-Seuk Lee, Jae Won Yoo, Seung-Hyun Kang, Yong Deog Hong, Song Seok Shin, Jae Youl Cho

**Affiliations:** ^1^Department of Genetic Engineering, Sungkyunkwan University, Suwon 440-746, Republic of Korea; ^2^Department of Food Science and Biotechnology, Sungkyunkwan University, Suwon 440-746, Republic of Korea; ^3^Medical Beauty Research Institute, AmorePacific R&D Center, Yongin 446-729, Republic of Korea; ^4^Sulloccha Research Center, Jangwon. Co., Ltd., Jeju 699-924, Republic of Korea; ^5^Cosmetics & Personal Care Research Division, Amorepacific R&D Center, Yongin 446-729, Republic of Korea

## Abstract

Lutein is a naturally occurring carotenoid with antioxidative, antitumorigenic, antiangiogenic, photoprotective, hepatoprotective, and neuroprotective properties. Although the anti-inflammatory effects of lutein have previously been described, the mechanism of its anti-inflammatory action has not been fully elucidated. Therefore, in the present study, we aimed to investigate the regulatory activity of lutein in the inflammatory responses of skin-derived keratinocytes or macrophages and to elucidate the mechanism of its inhibitory action. Lutein significantly reduced several skin inflammatory responses, including increased expression of interleukin-(IL-) 6 from LPS-treated macrophages, upregulation of cyclooxygenase-(COX-) 2 from interferon-**γ**/tumor necrosis-factor-(TNF-) **α**-treated HaCaT cells, and the enhancement of matrix-metallopeptidase-(MMP-) 9 level in UV-irradiated keratinocytes. By evaluating the intracellular signaling pathway and the nuclear transcription factor levels, we determined that lutein inhibited the activation of redox-sensitive AP-1 pathway by suppressing the activation of p38 and c-Jun-N-terminal kinase (JNK). Evaluation of the radical and ROS scavenging activities further revealed that lutein was able to act as a strong anti-oxidant. Taken together, our findings strongly suggest that lutein-mediated AP-1 suppression and anti-inflammatory activity are the result of its strong antioxidative and p38/JNK inhibitory activities. These findings can be applied for the preparation of anti-inflammatory and cosmetic remedies for inflammatory diseases of the skin.

## 1. Introduction

Inflammatory responses of the skin are largely due to infections with various bacteria and fungi, chemical irritation such as from sodium lauryl sulfate 2,4-dinitrophenol (DNP), and exposure of ultraviolet (UV) light, which lead to skin rash [1–4]. When such immunogens or irritants stimulate epithelial cells, macrophages, keratinocytes, mast cells, and Langerhans cells of the skin layer, various inflammatory mediators, including interferon-(IFN-) *γ*, tumor-necrosis-factor-(TNF-) *α*, interleukin-(IL-) 6, cyclooxygenase-(COX-) 2, and matrix metalloproteinases (MMP), are produced and evoke the symptoms of inflammation [5–7]. Activation of inflammatory cells and consequent expression of numerous inflammatory genes [8–11] result from toll-like-receptor-(TLR-) dependent [12, 13] or-independent stimulation of intracellular signaling cascades, which are composed of nonreceptor protein tyrosine kinases and serine-threonine protein kinases such as mitogen-activated protein kinases (e.g., p38, extracellular signal-regulated kinase [ERK], c-Jun N-terminal kinase [JNK]), as well as the activation and upregulation of nuclear factor (NF)-*κ*B and activator protein (AP)-1 transcription factors [14, 15].

**Figure 1:**
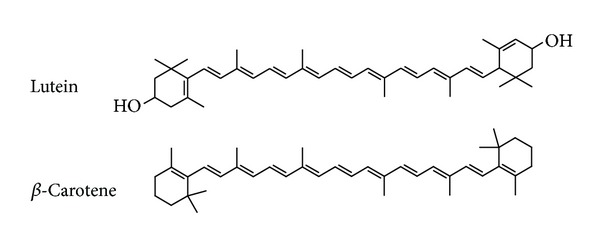
Chemical structure of lutein and *β*-carotene.

Lutein (Figure 1) is one of naturally occurring carotenoids with antioxidative, antitumorigenic, antiangiogenic, photoprotective, hepatoprotective, and neuroprotective properties [16–19]. Previous reports have suggested that this compound is able to ameliorate *in vitro* and *in vivo* inflammatory responses by suppressing NF-*κ*B activation [20, 21]. These findings strongly suggest a role of lutein in modulating inflammatory processes by regulating cellular redox potential. However, despite numerous studies, the mechanism underlying the anti-inflammatory activity of lutein remains unclear.

Because understanding skin inflammation is of interest to numerous cosmetic and pharmaceutical companies that develop skin-targeted biomaterials, exploring the effect of lutein on skin inflammation and its anti-inflammatory mechanism was undertaken. In the present study, the effect of lutein treatment on the expression of proinflammatory mediators in macrophages and keratinocytes treated with LPS, IFN-*γ*/TNF-*α*, and UV, and the action mechanism of lutein were carefully investigated.

## 2. Materials and Methods

### 2.1. Materials

Lutein (95% purity), *β*-carotene (Figure 1), 3-(4,5-dimethylthiazol-2-yl)-2,5-diphenyltetrazolium bromide (MTT), sodium nitroprusside (SNP), 2′7′-dichlorodihydrofluorescein diacetate (DCF-DA), and lipopolysaccharide (LPS; *E. coli* 0111:B4) were purchased from Sigma Chemical Co. (St. Louis, MO, USA). U0126 (U0), SB203580 (SB), and SP600125 (SP) were obtained from Calbiochem (La Jolla, CA, USA). Fetal bovine serum and RPMI 1640 were obtained from Gibco (Grand Island, NY, USA). The murine macrophage cell line RAW264.7 (ATCC No.: TIB-71) and the human keratinocyte cell line HaCaT (ATCC No.: HB-8065) were purchased from the ATCC (Rockville, MD, USA). All other chemicals were of analytical grade and were obtained from Sigma. Phosphospecific or total antibodies to AP-1 family proteins (c-Fos, c-Jun, and FRA-1), I*κ*B*α*, ERK, p38, JNK, MKK3/6, MKK4/7, TAK1, lamin A/C, and *β*-actin were obtained from Cell Signaling (Beverly, MA, USA). 

### 2.2. Cell Culture

RAW264.7 and HaCaT cells were cultured in RPMI 1640 medium supplemented with 10% heat-inactivated fetal bovine serum (FBS; Gibco, Grand Island, NY, USA), glutamine, and antibiotics (penicillin and streptomycin) at 37°C under 5% CO_2_. For each experiment, cells were detached with a cell scraper. When the cells were cultured for the experiments at 2 × 10^6^ cells/mL, the proportion of dead cells was less than 1% as determined by trypan blue dye exclusion.

### 2.3. Cell Viability Test

RAW264.7 and HaCaT cells (1 × 10^6^ cells/mL) were cultured for 18 h, after which lutein (0 to 50 *μ*M) was added to the cells for the final 24 h of culture. The cytotoxic effect of lutein was then evaluated by a conventional MTT assay, as reported previously [22, 23]. For the final 3 h of culture, 10 *μ*L MTT solution (10 mg/mL in phosphate-buffered saline, pH 7.4) was added to each well. The incubation was halted by the addition of 15% sodium dodecyl sulfate (SDS) into each well, which solubilized the formazan [24]. Absorbance at 570 nm (OD_570−630_) was measured using a SpectraMax 250 microplate reader (BioTex, Bad Friedrichshall, Germany).

### 2.4. Measurement of mRNA or Protein Levels of IL-6, COX-2, and MMP-9

RAW264.7 and HaCaT cells (1 × 10^6^ cells/mL) were cultured for 18 h, then pretreated with lutein (0 to 40 *μ*M) for 30 min, and further cultured with LPS (1 *μ*g/mL) or IFN-*γ* (20 ng/mL)/TNF-*α* (20 ng/mL) for 6 h or UV-irradiated for 27.5 s. The inhibitory effect of lutein on the expression of IL-6 and COX-2 was determined by semiquantitative and real-time quantitative RT-PCR [25, 26]. The protein levels of MMP-9 were detected by immunoblotting analysis. 

### 2.5. mRNA Analysis by Semiquantitative Reverse Transcriptase-Polymerase Chain Reaction (RT-PCR)

To determine cytokine mRNA expression levels, total RNA was isolated from LPS-treated RAW264.7 cells using TRIzol Reagent (Gibco BRL), according to the manufacturer's instructions. Total RNA was stored at −70°C until use. Semiquantitative (sq) or real-time quantitative (q) RT-PCR reactions were conducted as reported previously [27, 28]. The primers (Bioneer, Daejeon, Korea) used in these reactions are listed in Table 1.

### 2.6. Preparation of the Cell Lysates and Nuclear Fractions, Immunoblotting, and Immunoprecipitation

RAW264.7 or HaCaT cells (5 × 10^6^ cells/mL) were washed three times in cold PBS with 1 mM sodium orthovanadate, resuspended in lysis buffer (20 mM Tris-HCl, pH 7.4, 2 mM EDTA, 2 mM ethylene glycol tetraacetic acid, 50 mM *β*-glycerophosphate, 1 mM sodium orthovanadate, 1 mM dithiothreitol, 1% Triton X-100, 10% glycerol, 10 *μ*g/mL aprotinin, 10 *μ*g/mL pepstatin, 1 mM benzamide, and 2 mM PMSF), and lysed by sonication with rotation for 30 min at 4°C. The lysates were clarified by centrifugation at 16,000 ×g for 10 min at 4°C and stored at −20°C until use.

Nuclear lysates were prepared following a three-step procedure [29]. After treatment, cells were collected with a rubber policeman, washed with PBS, and lysed on ice for 4 min in 500 *μ*L lysis buffer containing 50 mM KCl, 0.5% Nonidet P-40, 25 mM HEPES (pH 7.8), 1 mM phenylmethylsulfonyl fluoride, 10 *μ*g/mL leupeptin, 20 *μ*g/mL aprotinin, and 100 *μ*M 1,4-dithiothreitol (DTT). Cell lysates were then centrifuged at 19,326 ×g for 1 min. For the second step, the nuclear fraction pellet was washed once in the washing buffer (identical to the lysis buffer described above, except without Nonidet P-40). In the final step, the nuclei were treated with an extraction buffer containing 500 mM KCl, 10% glycerol, and the other reagents listed for the lysis buffer above. The nuclei/extraction buffer mixture was frozen at −80°C, then thawed on ice, and centrifuged at 19,326 ×g for 5 min. The supernatant was collected as the nuclear extract. Soluble cell lysates were immunoblotted, and protein levels were visualized as previously reported [30]. For immunoprecipitation, cell lysates containing equal amounts of protein (500 *μ*g) from RAW264.7 cells (1 × 10^7^ cells/mL) treated with or without LPS (1 *μ*g/mL) for 2.5 min were precleared with 10 *μ*L protein A-coupled sepharose beads (50% v/v) (Amersham, Little Chalfont, Buckinghamshire, UK) for 1 h at 4°C. Pre-cleared samples were incubated with 5 *μ*L anti-p38 or JNK antibodies overnight at 4°C. Immune complexes were then mixed with 10 *μ*L protein A-coupled sepharose beads (50% v/v) and rotated for 3 h at 4°C.

### 2.7. p38 Enzyme Activity Assay

To determine the effect of lutein on LPS-activated p38 activity, immunoprecipitated p38 (prepared from RAW264.7 cells (5 × 10^6^ cells/mL) that had been treated with LPS for 30 min in the presence or absence of lutein) was incubated with ATF-2 according to the manufacturer's instructions. The p38 kinase activity was determined using an anti-phospho-ATF-2 antibody after immunoblotting analysis, as reported previously [31]. 

### 2.8. Neutralizing Activity of SNP-Derived Radicals and UV-Induced ROS

Lutein radicals scavenging activity was determined by measuring neutralizing activity of nitric oxide (NO^.^) released with SNP (20 mM) by spontaneous decomposition. The absorbance of the chromophore was measured at 540 nm. Percent inhibition of NO generation was measured by comparing the absorbance values of negative controls (10 mM sodium nitroprusside and vehicle) to assay preparations. For detection of reactive oxygen species (ROS) production, HaCaT cells were incubated with 50 *μ*M DCF-DA in culture medium for 30 min in a CO_2_ incubator. The cells were then rinsed with PBS to eliminate non-incorporated DCF-DA and treated with lutein, (*β*)-carotene, or vitamin C during UV-B exposure. The cells were imaged with a confocal laser-scanning microscope (Carl Zeiss, LSM510).

### 2.9. Statistical Analysis

**Figure 2:**
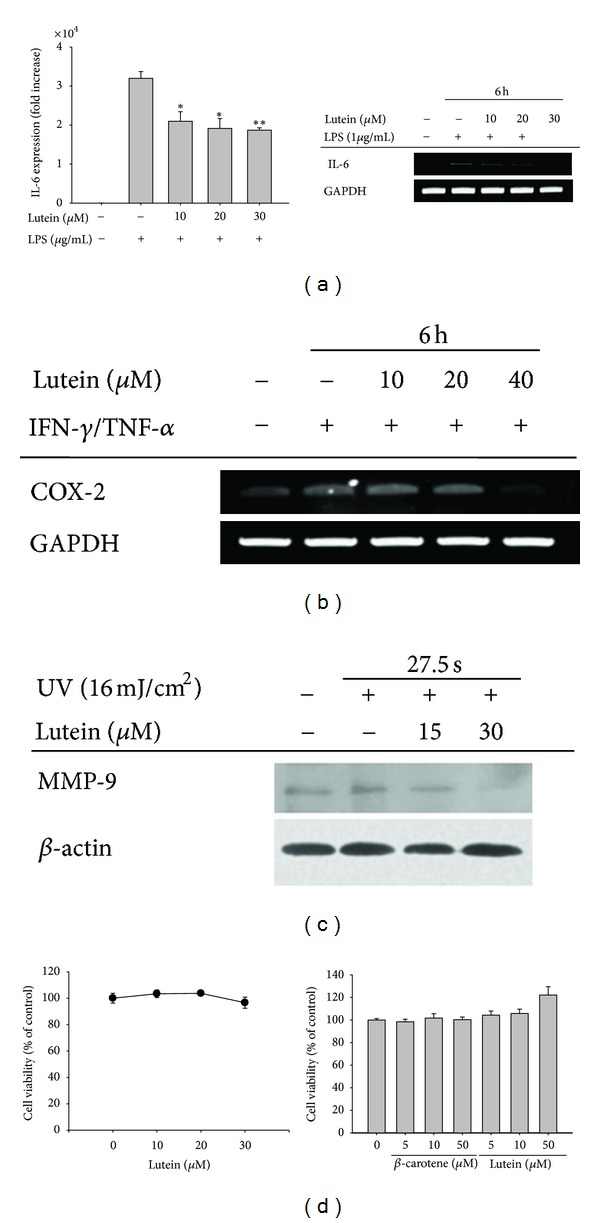
The effect of lutein on the expression of IL-6, COX-2, and MMP-9 in LPS-stimulated RAW264.7 cells, IFN-*γ*/TNF-*α*-treated HaCaT cells, and UV-irradiated HaCaT cells. (a) The level of IL-6 mRNA in RAW264.7 cells treated with lutein (0 to 30 *μ*M) in the presence or absence of LPS (1 *μ*g/mL) for 6 h was determined by real-time quantitative (right panel) or semiquantitative (left panel) RT-PCR. (b) The level of COX-2 mRNA in RAW264.7 cells treated with lutein (0 to 40 *μ*M) in the presence or absence of IFN-*γ* (20 ng/mL)/TNF-*α* (20 ng/mL) for 6 h was determined by semiquantitative RT-PCR. (c) The level of MMP-9 in HaCaT cells treated with lutein and UV-irradiated for 27.5 s was determined by immunoblotting analysis. (d) The viability of HaCaT cells was determined by MTT assays. *P < 0.05 and **P < 0.01 compared to the control.

Data (Figures 2(a), 2(d), and 4(a)) were expressed as the mean ± standard deviation (SD) as calculated from one (*n* = 6) of two independent experiments. Other data were representative of three different experiments with similar results. For statistical comparisons, results were analyzed using analysis of variance/Scheffe's post hoc test and the Kruskal-Wallis/Mann-Whitney test. All *P* values < 0.05 were considered statistically significant. All statistical tests were conducted using SPSS (SPSS Inc., Chicago, IL, USA).

## 3. Results and Discussion

Lutein is one of spontaneously generating carotenoids with anti-oxidative, anti-tumorigenic, anti-angiogenic, photoprotective, hepatoprotective, and neuroprotective properties [16–19]. Although the anti-inflammatory property of lutein has been suggested, the mechanism of lutein-mediated anti-inflammatory action in various skin inflammatory responses remains largely unclear. Therefore, in the present study, we aimed to elucidate the anti-inflammatory activity of lutein and its inhibitory mechanism by mimicking skin inflammatory conditions. 

First, the ability of lutein to attenuate inflammatory responses in macrophages and skin-derived keratinocytic (HaCaT) cells during various pro-inflammatory conditions induced by LPS, IFN-*γ*/TNF-*α*, and UV-irradiation [7, 32] was examined. Interestingly, this compound significantly suppressed the expression of IL-6 mRNA, a major cytokine involved in skin inflammation [33], as determined by quantitative (Figure 2(a), left panel) or semiquantitative (Figure 2(a), right panel) RT-PCR. Under the conditions, however, up to 30 *μ*M of lutein exhibited no cytotoxic activity in RAW264.7 cells (Figure 2(d) left panel). In addition, lutein suppressed the expression of COX-2 induced by cotreatment with IFN-*γ* and TNF-*α* (Figure 2(b)), indicating that this compound is able to block the production of inflammatory mediators in the skin. Moreover, this compound also suppressed MMP-9 expression triggered by UV irradiation (Figure 2(c)) without altering the viability of HaCaT cells (Figure 2(d) right panel), indicating that lutein is also able to protect against UV irradiation-mediated skin irritation. It has been previously reported that lutein can decrease the edematous cutaneous response as illustrated by the reduction of the UVB-induced increase of ear bifold thickening [34], that aromatic carotenoids can prevent UV-induced DNA damage in human skin fibroblasts [35], and that lutein can suppress melanogenesis [36]. In agreement with these studies, our data further confirm that lutein can be used as a skin protective agent with anti-inflammatory functions. 

**Figure 3:**
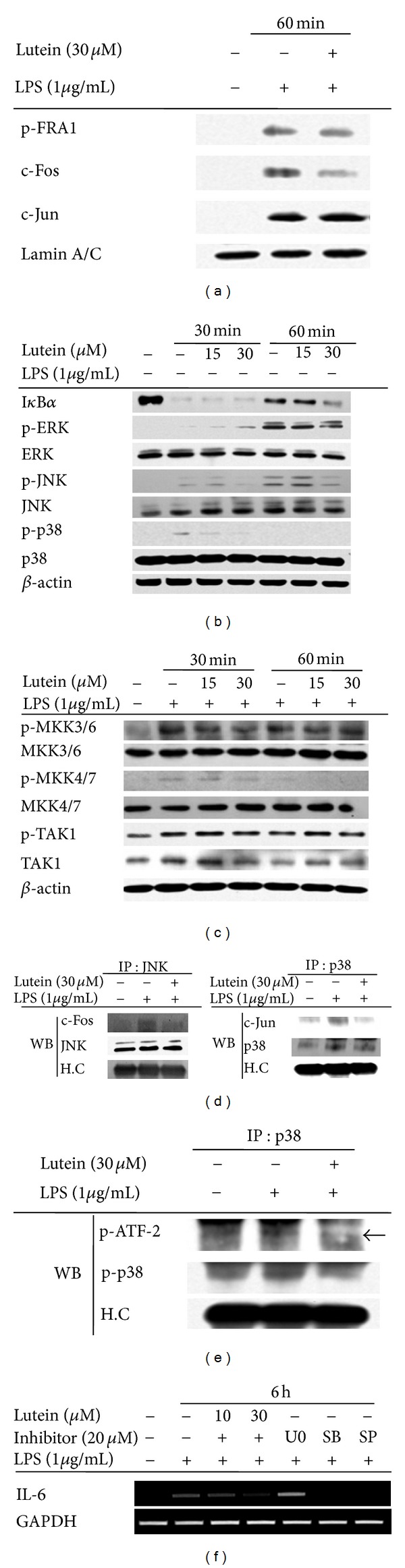
The effect of lutein on the activation of AP-1 and its upstream signaling cascades. (a) The levels of AP-1 family proteins, p-FRA-1, c-Fos, and c-Jun in the nuclear fraction were determined by immunoblotting analyses using antibodies against phospho- or total proteins. ((b) and (c)) Phosphoprotein or total protein levels of I*κ*B*α*, p38, ERK, JNK, MKK3/6, MKK4/7, TAK1, and *β*-actin from cell lysates were determined by immunoblotting analyses using phospho-specific or total protein antibodies. (d) An interaction between JNK and c-Fos or p38 and c-Jun was evaluated by immunoprecipitation and immunoblotting analyses. RAW264.7 cells (5 × 10^6^ cells/mL) were incubated with lutein (30 *μ*M) in the presence or absence of LPS (1 *μ*g/mL) for 30 min. c-Jun or c-Fos was immunoprecipitated from whole cell lysates using a specific antibody to JNK or p38, followed by immunoblotting with antibodies to c-Fos, JNK, c-Jun, and p38, as well as rabbit immunoglobulin heavy chain. (e) The kinase activity of immunoprecipitated p38 prepared from LPS-treated RAW264.7 cells was determined by measuring the level of phospho-ATF-2. The level of phosphorylated ATF-2 was measured by immunoblotting analysis. (f) The level of IL-6 mRNA from RAW264.7 cells treated with lutein (0 to 30 *μ*M) or enzyme inhibitors (U0126 (U0), SB203580 (SB), or SP600125 (SP)) in the presence or absence of LPS (1 *μ*g/mL) for 6 h was determined by semiquantitative RT-PCR. Relative intensity was calculated by densitometric scanning.

Furthermore, to evaluate the inhibitory activity of lutein on the expression of pro-inflammatory genes, we examined the inflammatory signaling pathways and corresponding transcription factors activated in response to pro-inflammatory stimuli (e.g., LPS). Interestingly, we determined that the inhibition of IL-6 mRNA expression by lutein was a result of the suppression of nuclear translocation of p-FRA and c-Fos (Figure 3(a)), major components of AP-1 family [37]. Although NF-*κ*B has been previously reported to be a major target transcription factor [21, 38], we did not observe a strong inhibitory pattern of p65/p50 translocation in response to LPS (data not shown), and the degradation level of I*κ*B*α* was not restored by lutein treatment (Figure 3(b)). Because the activation of AP-1 is known to be mediated by the phosphorylation of MAPK (p38, ERK, and JNK) [39], we next examined whether lutein is capable of reducing the levels of the phosphoproteins. Intriguingly, this compound was found to clearly suppress the phosphorylation of p38 and JNK at 15 to 30 *μ*M (Figure 3(b)). Furthermore, the phosphorylation of MKK3/6 and MKK4/7 but not TAK1 at 30 min and MKK4/7 at 60 min was strongly diminished by 30 *μ*M of lutein (Figure 3(c)). The formation of signaling complex composed of JNK and c-Fos or p38 and c-Jun (Figure 3(d)) and kinase activity of p38 upregulated by LPS (Figure 3(e)) were also remarkably reduced by this compound, implying that suppression of p38 and JNK phosphorylation pathways might negatively affect the molecular interaction between MAPK (p38 and JNK) and AP-1 family proteins. In agreement with this finding, the inhibitors (SP600125 (SP) and SB203580 (SB)) of JNK and p38, as well as lutein (10 and 30 *μ*M), strongly reduced the expression of IL-6 (Figure 3(f)), suggesting a role of JNK- and p38-mediated signaling cascade in IL-6 expression. Although lutein has not been reported to modulate AP-1 activation signaling, structural derivatives, such as lycopene, *β*-carotene, or *β*-cryptoxanthin, were considered as AP-1 regulatory compounds [40–42]. Thus, these data strongly indicate a role of lutein in negative regulation of AP-1-mediated inflammatory gene expression.

**Figure 4:**
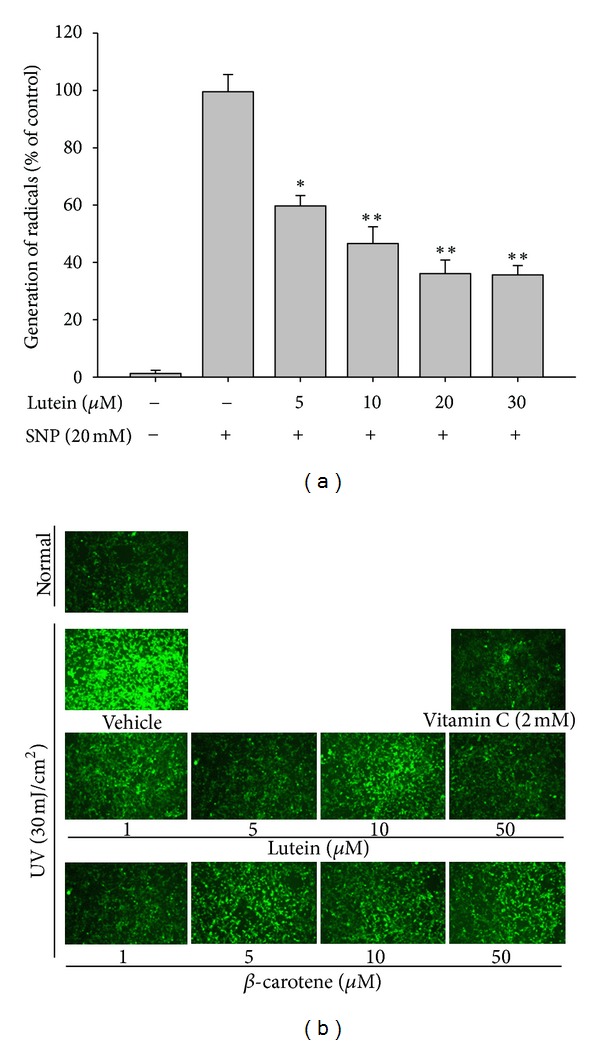
The scavenging effect of lutein on the generation of radicals and ROS in UV-irradiated HaCaT cells. (a) The radical scavenging activity of lutein was determined by measuring the levels of NO^.^ released from SNP (20 mM) in the presence or absence of lutein. (b) Immediately after UVA exposure, ROS production was quantified by measuring the fluorescence from the oxidation product of carboxy-H_2_DCF-DA, as described in Section 2. *P < 0.05 and **P < 0.01 compared to the control.

How this compound can interrupt p38/AP-1 pathway activated by LPS is not clear yet in this study. However, because AP-1 pathway is an important inflammatory signaling pathway activated by intracellular ROS [43], we next evaluated the antioxidative activity of lutein in blocking AP-1 activation by suppressing ROS generation as an approach of mechanistic understandings. As predicted, this compound strongly neutralized SNP-induced radical generation (Figure 4(a)). Similarly, noncytotoxic concentrations of lutein (Figure 2(d)) also dramatically scavenged the elevated ROS generated by UV irradiation (Figure 4(b)), indicating that UV irradiation-mediated cellular responses can be reverted by lutein treatment. Indeed, this compound strongly suppressed the expression of MMP-9 (Figure 2(c)), a marker of acute inflammation [44], implying that lutein is capable of modulating UV-mediated inflammatory and cellular damage by suppressing radical generation. In addition, lutein has been reported to reduce oxidative stress induced by benzo(a)pyrene [45], hypercholesterolemic diet [46], H_2_O_2_ [47], and D-galactose [48]. Taken together, these prior reports and our new data suggest that the radical scavenging activity of lutein is a common feature observed in lutein pharmacology.

In summary, our findings demonstrate that lutein strongly inhibits several skin inflammatory responses such as expression of IL-6, COX-2, and MMP-9 from LPS-treated macrophages, IFN-*γ*/TNF-*α*-stimulated HaCaT cells, and UV-irradiated keratinocytes. By examining the intracellular signaling cascade and the nuclear levels of transcription factor, we demonstrate that lutein can suppress the activation of redox-sensitive AP-1 pathway. Based on the radical and ROS scavenging activity of lutein, it was concluded that the AP-1-targeted anti-inflammatory activity of lutein was due to its anti-oxidative activity. Therefore, our results strongly suggest that due to its anti-oxidative properties, lutein can be used as an anti-inflammatory and cosmetic remedy for inflammatory diseases of the skin. 

## Figures and Tables

**Table 1:** Primer sequences used in the RT-PCR analysis.

Gene	Primer sequences
IL-6 (sqPCR)	
F	5′-GTACTCCAGAAGACCAGAGG-3′
R	5′-TGCTGGTGACAACCACGGCC-3′
IL-6 (qPCR)	
F	5′-AACGATGATGCACTTGCAGA-3′
R	5′-GAGCATTGGAAATTGGGGTA-3′
COX-2 (sqPCR)	
F	5′-CACTACATCCTGACCCACTT-3′
R	5′-ATGCTCCTGCTTGAGTATGT-3′
GAPDH (sqPCR)	
F	5′-CACTCACGGCAAATTCAACGGCAC-3′
R	5′-GACTCCACGACATACTCAGCAC-3′
